# Evidence supporting cryptic species within two sessile microinvertebrates, *Limnias melicerta* and *L*. *ceratophylli* (Rotifera, Gnesiotrocha)

**DOI:** 10.1371/journal.pone.0205203

**Published:** 2018-10-31

**Authors:** Azar Kordbacheh, Robert L. Wallace, Elizabeth J. Walsh

**Affiliations:** 1 Department of Biological Sciences, University of Texas at El Paso, El Paso, Texas, United States of America; 2 Department of Biology, Ripon College, Ripon, Wisconsin, United States of America; University of Minnesota, UNITED STATES

## Abstract

Microorganisms, including rotifers, are thought to be capable of long distance dispersal. Therefore, they should show little population genetic structure due to high gene flow. Nevertheless, substantial genetic structure has been reported among populations of many taxa. In rotifers, genetic studies have focused on planktonic taxa leaving sessile groups largely unexplored. Here, we used COI gene and ITS region sequences to study genetic structure and delimit cryptic species in two sessile species (*Limnias melicerta* [32 populations]; *L*. *ceratophylli* [21 populations]). Among populations, ITS region sequences were less variable as compared to those of the COI gene (ITS; *L*. *melicerta*: 0–3.1% and *L*. *ceratophylli*: 0–4.4%; COI; *L*. *melicerta*: 0–22.7% and *L*. *ceratophylli*: 0–21.7%). Moreover, *L*. *melicerta* and *L*. *ceratophylli* were not resolved in phylogenetic analyses based on ITS sequences. Thus, we used COI sequences for species delimitation. Bayesian Species Delimitation detected nine putative cryptic species within *L*. *melicerta* and four putative cryptic species for *L*. *ceratophylli*. The genetic distance in the COI gene was 0–15.4% within cryptic species of *L*. *melicerta* and 0.5–0.6% within cryptic species of *L*. *ceratophylli*. Among cryptic species, COI genetic distance ranged 8.1–21.9% for *L*. *melicerta* and 15.1–21.2% for *L*. *ceratophylli*. The correlation between geographic and genetic distance was weak or lacking; thus geographic isolation cannot be considered a strong driver of genetic variation. In addition, geometric morphometric analyses of trophi did not show significant variation among cryptic species. In this study we used a conservative approach for species delimitation, yet we were able to show that species diversity in these sessile rotifers is underestimated.

## Introduction

Microorganisms are capable of long distance dispersal thus it has been suggested that they have cosmopolitan distributions with little geographic structure [1–4]. However, biogeographical patterns have been documented for many taxa such as soil [5] and marine bacteria [6–8], protists [9,10], fungi [11], and rotifers [12,13]. One of the reasons that microorganisms are often considered ubiquitous is the failure to identify cryptic species [14,15]. However, cryptic species complexes commonly occur in these taxa (e.g., [16–19]).

Similar to the other microorganisms, rotifers have passive dispersal through their dormant stages [20,21]. Thus, researchers inferred that rotifers have cosmopolitan distributions (e.g., [22]), and thus should show little population genetic structure due to gene flow. However, incorporating molecular tools in the study of rotifer diversity has provided ample evidence of cryptic species complexes and substantial genetic structure within and among rotifer populations (e.g., [23–30]). By definition cryptic species are not readily distinguished by morphology, but subtle morphological variations have been detected among species in some complexes. For instance, size and shape of lorica varies among cryptic species of *Brachionus plicatilis* Müller, 1786 (e.g., [31–36]), trophi and resting egg morphology differ in the *Epiphanes senta* (Müller, 1773) complex [37], and trophi size varies among populations of *Rotaria magnacalcarata* (Parsons, 1892) [38].

Genetic differentiation and the rate of diversification may vary among aquatic taxa with different life histories [39–41] and/or habitat differences [42]. Rotifer species can be planktonic, associated with littoral vegetation, or sessile [20]. In littoral zones there is higher habitat diversity provided by vegetation, debris, mosses, and filamentous algae as compared to the pelagic zone [20]. Habitat preference has been reported for some rotifers inhabiting littoral zones such as *Collotheca campanulata* (Dobie, 1849) [43–44] and *Euchlanis dilatata* Ehrenberg, 1830 [44]. Difference in habitat preference may limit connectivity among populations and cause genetic divergence [45]. Therefore, as a result of habitat differentiation, substantial genetic diversity is expected within and among populations [46]. In addition, genetic divergence among populations may be related to dispersal capabilities. For example, Russo *et al*. [47] found that genetic variation in 16 allozyme markers within two anemone species, *Bunodosoma caissarum* Correa, 1987 and *Actinia bermudensis* McMurrich, 1889 was related to their dispersal ability. *B*. *caissarum* has a long planktonic larval period that provides high dispersal capability. This species showed lower genetic structure compared to that of *A*. *bermudensis* with low dispersal abilities. Similarly, Lee *et al*. [48] found high genetic divergence among populations of the jelly *Rhizostoma octopus* (Linnaeus 1788) with bipartite life history (Φ_ST_ ≤ 0.75) and Stopar *et al*. [49] found low genetic differentiation within the holoplanktonic jelly *Pelagia noctiluca* (Forskål 1775) (Φ_ST_ ≤ 0.09) in the COI gene. Genetic differentiation within these cnidarian species may be associated with the variation in their dispersal capabilities. Therefore, sessile rotifers may show higher genetic structure than planktonic groups because in sessile rotifers females are mobile only during their larval stage, which can limit their dispersal range. However, similar to non-sessile rotifers, their resting stage may be transported across long distances.

Almost all studies on genetic structure and cryptic species of monogonont rotifers focus on planktonic taxa, with little attention having been paid to sessile species. Sessile rotifers of orders Collothecaceae and Flosculariaceae from superorder Gnesiotrocha are common in a wide assortment of aquatic habitats and attach to varied substrata [50]. Study of these forms is challenging as their plant substrata must be inspected to find them and several diagnostic characteristics need to be examined in live individuals [20]. Thus, they are often overlooked, which has led to gaps in our knowledge about their taxonomic diversity. Molecular tools have been applied in few phylogenetic studies that have included sessile rotifers (e.g., [51,52]), but these have not focused on examination of population level genetic patterns or detection of cryptic species.

One poorly studied sessile taxon is the genus *Limnias* (Flosculariidae), in which six morphospecies are currently recognized [53,54]: *Limnias ceratophylli* Schrank, 1803; *L*. *cornuella* Rousselet, 1889; *L*. *melicerta* Weisse, 1848, *L*. *myriophylli* (Tatum, 1868), *L*. *nymphaea* Stenroos, 1898, and *L*. *shiawasseensis* Kellicott, 1888. *Limnias melicerta* and *L*. *ceratophylli* are considered cosmopolitan and each has been reported from seven biogeographical regions [43]. The other four species have restricted distributions. *Limnias cornuella* has only been reported from Palearctic [55], *L*. *myriophylli* is reported from Afrotropical and Palearctic [43], *L*. *nymphaea* from Palearctic, and *L*. *shiawasseensis* from Nearctic biogeographical regions [43]. The few available studies on this genus include the following: taxonomy [54–58], trophi descriptions [51,59], tube formation [58], tube ultrastructure of *L*. *melicerta* [60], post-natal development [61], phylogeny of Flosculariaceae [51], ecological studies (i.e., water quality and abundance (*L*. *melicerta*; [62,63]), population growth (*L*. *melicerta* and *L*. *ceratophylli*; [64]), and toxicology [65]). To our knowledge, there are no published studies on genetic population structure within or among species of this genus.

We hypothesize that sessile rotifers will have a discernable genetic structure that will be more pronounced than that of planktonic rotifers and that this structure is sufficient to define independently evolving lineages as cryptic species. To test these hypotheses we studied genetic structure, identified cryptic species, and investigated geographic isolation of genotypes in *Limnias melicerta* and *L*. *ceratophylli*. We used partial mitochondrial *cytochrome c oxidase subunit I* (COI) gene and the internal transcribed spacer (ITS) region sequences from a broad geographic range. Partial 18S rRNA sequences were used to confirm monophyly of the two species. In addition, we examined morphological variation in trophi among putative cryptic species within *L*. *melicerta* and *L*. *ceratophylli*.

## Materials and methods

### Sample collection and culture

Aquatic plant samples were collected from habitats across the USA and a sediment sample from Australia (S1 and S2 Tables). *Limnias melicerta* and *L*. *ceratophylli* were identified and isolated from rehydrated sediments or by removing a piece of vegetation to which they were attached. Species identification was based on tube structure, shape of corona, antennae length, and the number of dorsal nodules [55]. Clonal lineages initiated from single females were cultured in modified MBL media [66] and fed a mixture of the algae *Chlorella vulgaris* Berijerinck, 1890 (The UTEX Culture Collection of Algae at the University of Texas at Austin [UTEX] strain 30) and *Chlamydomonas reinhardtii* Dangeard, 1888 (UTEX strain 90). Rotifers in this genus produce tubes of hardened secretions [20]; we added powdered carmine (Alfa Aesar, UK) to lab cultures to provide a supplementary matrix to aid tube construction and to increase their visibility in culture.

Voucher specimens were deposited in the UTEP Biodiversity Collections at The University of Texas at El Paso (*L*. *melicerta*: UTEP:Zoo:43, 105–134; *L*. *ceratophylli*: UTEP:Zoo:32–42, UTEP:Zoo:52–61). Deposited specimens included approximately 10 individuals from each population preserved in 95% ethanol and 10 clonal individuals preserved in 4% buffered formalin for molecular analyses and identification, respectively.

### DNA extraction and gene amplification

DNA was extracted from one individual of each clonal lineage by adding 13 μl Chelex-100 (*Bio*-*Rad* Laboratories, CA, USA) and incubating at 100°C for 10 min. DNA templates were stored at -80°C until used for amplification. Number of clonal lineages examined from each population is given in S1 and S2 Tables.

An approximate 630 bp portion of the *cytochrome c oxidase subunit I* (COI) gene was amplified using the primers LCO1490: 5' -GGTCAACAAATCATAAAGATATTGG-3' and HCO2198: 5'-TAAACTTCAGGGTGACCAAAAAATCA-3' [67]. The entire nuclear internal transcribed spacer region (ITS) was amplified using the primers ITS4: 5'-TCCTCCGCTTATTGATATGC-3' and ITS5: 5'-GGAAGTAAAAGTCGTAACAAGG-3' [68], and 865 bp of the 18S rRNA gene was amplified using primers 3F: 5’-GTTCGATTCCGGAGAGGG-3’ as modified by Giribet *et al*. [69] and primer 18Sbi: 5’-CTAGAGTCTCGTTCGTTATCGG-3’ as modified by Whiting *et al*. [70].

PCR reactions contained 10 μl of genomic DNA, 1 μl of each primer (500 ng/ μl), 22 μl HPLC grade sterile water, 1 μl GoTaq G2 DNA Polymerase (Promega), 10 μl 5X PCR buffer B (10 mM MgCl_2_, pH 8.5, Invitrogen) or 5X PCR buffer A (7.5 mM MgCl_2_, pH 8.5, Invitrogen), followed by adding 5 μl dNTP mix (2.5 mM each of dATP, dCTP, dGTP, dTTP) at 80°C. PCR cycles were run on a thermocycler (Techne TC-412) and consisted of an initial denaturation at 94°C for 1 min, followed by denaturation at 94°C for 1 min, annealing at 48°C for 2 min and extension at 72°C for 3 min for 35 cycles, and a final extension step at 72°C for 7 min. To verify the size of amplification products we used electrophoresis, and we purified them using GENECLEAN kits (MP Biomedicals, LLC) before sequencing. Sequencing was done at UTEP’s BBRC Genomic Analysis Core Facility on an Applied Biosystems 3130xl Genetic Analyzer using BigDye Terminator v3.1 Cycle Sequencing Kits (Applied Biosystems). GenBank accession numbers for all sequences obtained are given in S1 Table (*L*. *melicerta*) and S2 Table (*L*. *ceratophylli*). The COI gene sequences of *L*. *melicerta* (accession number, KT870155.1) and *L*. *ceratophylli* (KT870157.1) from GenBank are not included in our analyses for two reasons. 1) The COI sequence of *L*. *melicerta* KT870155.1 is 330 bp, which was much shorter than COI sequences obtained in this study (623 bp). 2) The COI sequence of *L*. *ceratophylli* KT870157.1 grouped with cryptic species M of *L*. *melicerta* in phylogenetic analyses. Additional 18S rRNA sequences (*L*. *melicerta*: KM873599.1, *L*. *ceratophylli*: KM873598.1) and a COI sequence from *L*. *melicerta* (KT870154.1) were included from GenBank. *Sinantherina socialis* (Linneaus, 1758) and *Ptygura pilula* (Cubitt, 1872) were included as outgroups in phylogenetic analyses for the COI gene. *Floscularia conifera* (Hudson, 1886) and *Ptygura brachiata* (Hudson, 1886) were used as outgroup taxa in phylogenetic analyses based on ITS region. For analysis of 18S rRNA sequences, we used *Collotheca campanulata* as the outgroup taxon (S1 Table).

### Genetic diversity

FinchTV v 1.4.0 [71] was used to check sequences manually, especially for potential double peaks in the ITS region sequences. The ITS region alignment was uploaded to the SeqPhase online tool (http://seqphase.mpg.de/seqphase/) to phase the sequences as described by Flot [72]. Contigs for all sequences were made using CAP 3 [73] and were aligned using MAFFT v 7 [74]. Mesquite v 3.2 [75] was used to manually check the alignments and to translate COI gene sequences to proteins. To measure substitution saturation, we used DAMBE v 6 [76]. Number of polymorphic sites, number of parsimony informative sites, number of haplotypes, haplotype diversity (*h*), and nucleotide diversity (*π*) were calculated using DnaSp v 5.10.01 [77], and uncorrected pairwise sequences distances ("p") were calculated in Mega v 7.0 [78]. A haplotype network was constructed using the median joining method in Network v 5.0.3 [79].

### Species delimitation

Models for sequence evolution were TPM2uF+I+G for the COI gene, TPM1uF+I for the ITS region, and JC for the 18S rRNA gene as determined using Jmodeltest2 [80,81] available at the CIPRES Science Gateway 3.3 [82]. To construct the phylogenetic trees, Bayesian analysis was run for 10^7^ generations with two parallel runs and a 25% burn-in period using MrBayes v 3.2.6 on XSEDE high-throughput computing resources available at CIPRES Science Gateway [82]. Phylogenetic analyses were implemented in BEAST and *BEAST [83] using GTR+I+G model of sequence evolution for the COI gene and GTR+I model for the ITS region. TPM2uF and TPM1uF models are not available in BEAST. However, both of these models are classified under the GTR model. Thus GTR was used in both instances.

To determine the number of evolutionary entities (putative cryptic species), we used Generalized Mixed Yule Coalescent (GMYC, [84]), Poisson Tree Process (PTP, [85]), Automatic Barcoding Gap Discovery (ABGD, [86]), *BEAST v 1.8.3 [83], and Bayesian Species Delimitation (BSD) implemented in Bayesian Phylogenetics and Phylogeography software (BPP v 3.1, [87–89]).

We used BEAST v 1.8.3 [83] to construct ultrametric trees and *BEAST v 1.8.3 [83] for species delimitation. Both analyses were run for the COI gene and ITS region sequences separately for 10^7^ generations, with sampling every 1,000 generations. Tracer v 1.6.0 [90] was used to check the effective sample size (ESS>200) and to verify convergence. Consensus trees were obtained using TreeAnnotator v 1.8.3 with a 25% burn-in. Ultrametric trees were used for species delimitation in single threshold and multiple threshold GMYC [91] (http://species.h-its.org/gmyc/, accessed June 12, 2018), Bayesian GMYC (bGMYC) [92] and PTP methods. bGMYC was run using the R package *bGMYC* v 1.0.2 for 100,000 iterations with sampling every 1,000 iterations. We ran PTP by uploading the ultrametric trees to the online tool available at http://species.h-its.org/ptp/ (accessed June 12, 2018) and used default settings. ABGD delimitation was done by uploading the sequence alignment to the online tool available at www.abi.snv.jussieu.fr/public/abgd/ under the default settings (accessed June 12, 2018).

*BEAST v 1.8.3 [83] was run under assumptions regarding the number of species for both the COI gene and ITS region sequences. Lineages having posterior probabilities > 0.90 were retained in *BEAST analyses. To run BSD, the phylogenetic tree based on Bayesian inference was used as the guide tree, and we used the joint species delimitation and tree estimation method (unguided species delimitation) that does not rely on the topology of the guide tree.

### Isolation by distance

Geographic distance matrices were constructed using Geographic Distance Matrix Generator v 1.2.3 [93]. To test the correlation between genetic variation and geographic distances (log transformed; km) among populations, Mantel tests with 10,000 permutations were run using the R package *ecodist* v 1.2.9 [94].

### Trophi morphology

Trophi were prepared for scanning electron microscopy (SEM) by dissolving rotifer tissue in ~5% sodium hypochlorite, rinsing with deionized water 10–15 times, and air-drying on circular cover slips at room temperature [95]. Trophi were coated with gold/palladium using a Gatan 682 PECS sputter coater. SEM images were obtained at 20 kV using a Hitachi S-4800 system. Trophi were prepared for individuals from one clonal lineage from each examined population.

**Fig 1:**
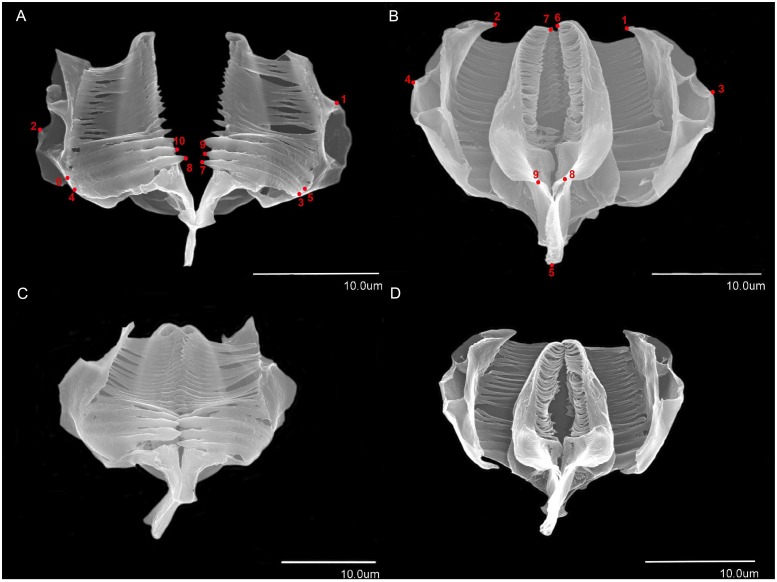
Shape of trophi and landmarks used in geometric morphometric analyses. A: frontal view of *Limnias ceratophylli* trophi, 1, 2: midpoint of manubrium, 3, 4: base of first large tooth of ramus, 5, 6: base of second large tooth of ramus, 7–8: tip of first large tooth of ramus, 9, 10: tip of second large tooth of ramus. B: caudal view of *L*. *ceratophylli* trophi, 1, 2: apical point of manubrium, 3, 4: midpoint of manubrium, 5: apical point of fulcrum, 6, 7: apical point of ramus, 8, 9: basal point of ramus. C: frontal view of *Limnias melicerta* trophi. D: caudal view of *L*. *melicerta* trophi.

We used a geometric morphometric approach to study variation in the shape and size of 92 trophi for *L*. *melicerta* and 60 trophi for *L*. *ceratophylli* among putative cryptic species. This method uses Cartesian coordinates for a set of anatomical landmarks [96]. SEM images were obtained from caudal and frontal views of the trophi. Using TPS series software [97], nine landmarks on the caudal view and 10 on the frontal view of the trophi were digitized (Fig 1). Configuration of landmarks were analyzed using Generalized Procrustes Analysis [96]. Trophi size was calculated as Centroid Size (CS): i.e., the square root of the sum of squared distances between landmarks and their centroid [98]. Variation in the shape of trophi based on landmarks was examined using Discriminant Analysis in SPSS v 24 [99]. Because trophi centroid size was not normally distributed, variation in size among putative cryptic species was tested using a non-parametric Kruskal-Wallis test, and between the two morphospecies using a non-parametric Mann-Whitney U test implemented in SPSS v 24 [99].

Methods used in this study are available at protocols.io, dx.doi.org/10.17504/protocols.io.tppemmn.

## Results

### Genetic diversity

For the COI gene, sequences were analyzed for 72 individuals from *Limnias melicerta* and 25 individuals from *L*. *ceratophylli*. The ITS region was sequenced for 76 individuals from *L*. *melicerta* and 35 individuals from *L*. *ceratophylli*. For 18S rRNA, sequences were acquired for 17 individuals of *L*. *melicerta* and 18 individuals of *L*. *ceratophylli*. Alignment length was 623 bp for the partial COI gene sequences, 763 bp for the ITS region including insertions, and 865 bp for partial 18S rRNA gene sequences. COI gene, ITS region, and 18S rRNA sequences were not saturated (index of substitution saturation < critical index of substitution saturation, p < 0.001, S3 Table). For the COI gene, haplotype diversity was 0.87 for *L*. *melicerta* and 0.94 for *L*. *ceratophylli*; nucleotide diversity was 0.13 for *L*. *melicerta* and 0.11 for *L*. *ceratophylli*. The overall genetic distance in the COI gene among populations was 0–22.7% for *L*. *melicerta* and 0–21.7% for *L*. *ceratophylli*, and the genetic distance between *L*. *melicerta* and *L*. *ceratophylli* ranged from 19.2 to 24.1%. For the ITS region, haplotype diversity was 0.67 for *L*. *melicerta* and 0.56 for *L*. *ceratophylli*, nucleotide diversity was 0.01 for *L*. *melicerta* and 0.02 for *L*. *ceratophylli*. The overall genetic distance in the ITS region among populations was 0–3.1% for *L*. *melicerta* and 0–4.4% for *L*. *ceratophylli*, and the genetic distance between *L*. *melicerta* and *L*. *ceratophylli* was 1.2–5.6%. We also found seven heterozygous individuals for the ITS region in *L*. *melicerta* and two in *L*. *ceratophylli*. Based on 18S rRNA sequences, haplotype diversity, nucleotide diversity, and genetic diversity were 0 for both *L*. *melicerta* and *L*. *ceratophylli*. The genetic distance (uncorrected “p” distance) between *L*. *melicerta* and *L*. *ceratophylli* was 0.5%. Genetic diversity measures for all markers are summarized in Table 1.

**Table 1:** Summary of genetic measures for COI gene, ITS region, and partial 18S rRNA sequences.

	*Limnias melicerta*			*Limnias ceratophylli*		
	COI gene	ITS region	18S rRNA	COI gene	ITS region	18S rRNA
Inter-population genetic variation (%)	0.3–22.7	0–3.1	0	0–21.7	0–4.4	0
Intra-population genetic variation (%)	0–0.8	0	ND	0–1.9	0	ND
Number of haplotypes/ number of sequences	31/72	12/76	1/18	15/25	4/35	1/18
Haplotype diversity	0.87	0.67	0	0.94	0.56	0
Nucleotide diversity	0.13	0.01	0	0.11	0.02	0
Polymorphic sites/ number of base pairs	249/623	47/763	0/865	193/623	54/763	0/865
Parsimony informative sites	241	24	0	148	43	0
Heterozygous individuals (#)	NA	7	NA	NA	2	NA

Inter- and intra-population genetic variation (uncorrected “p” distance), haplotype and nucleotide diversity, number of haplotypes, number of polymorphic sites for partial COI gene, ITS region, and partial 18S rRNA sequences, and the number of heterozygotes detected by phasing ITS region in *Limnias melicerta* and *L*. *ceratophylli* populations surveyed. NA = Not applicable. ND = No data.

### Species delimitation

**Fig 2:**
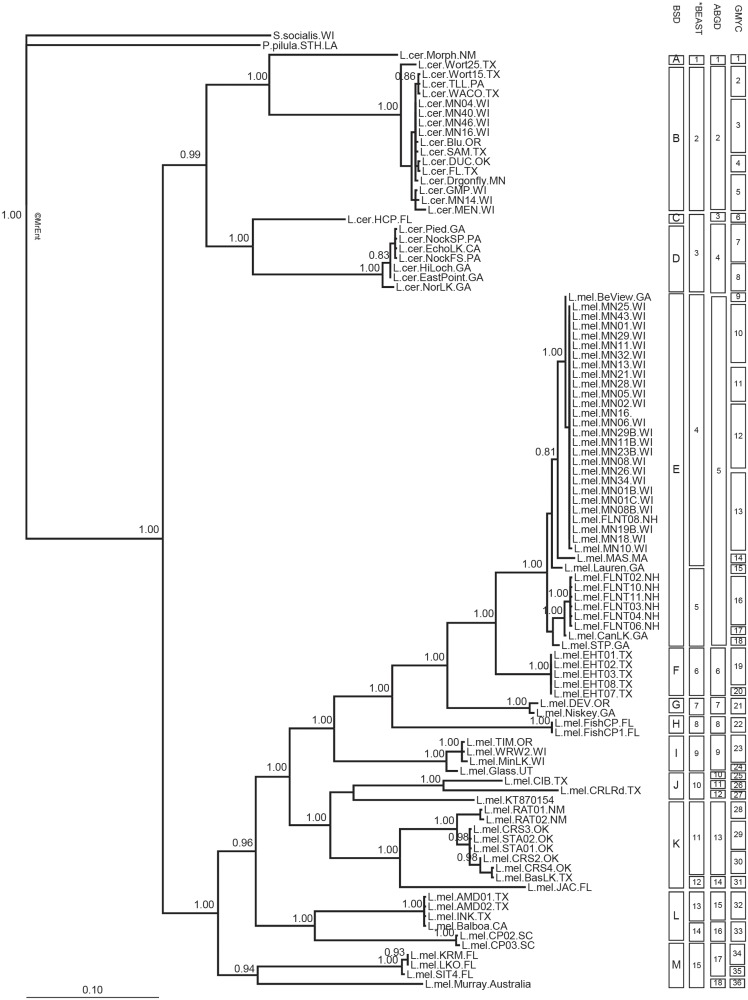
Bayesian inference consensus phylogenetic tree based on partial COI gene sequences of 32 populations of *Limnias melicerta* and 21 populations of *L*. *ceratophylli*. Average branch lengths are proportional to the number of substitutions per site under a TPM2uF+I+G substitution model. At each node posterior probabilities > 0.80 are shown. Putative cryptic species detected using Bayesian Species Delimitation (BSD), *BEAST, Automatic Barcoding Gap Discovery (ABGD), and Single Threshold Generalized Mixed Yule Coalescent models (GMYC) are shown. Abbreviations as in S1 and S2 Tables; independent clonal isolates are indicated by a number (e.g. 01).

**Fig 3:**
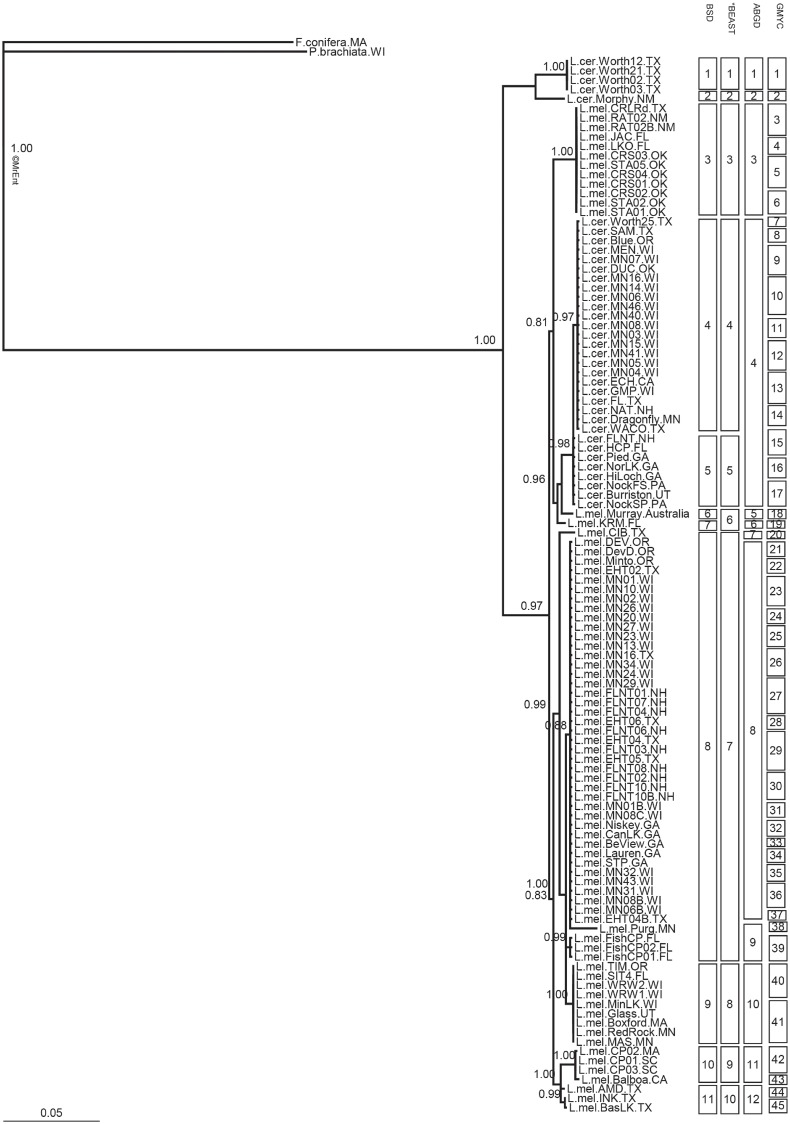
Bayesian inference consensus phylogenetic tree based on ITS region sequences of 37 populations of *Limnias melicerta* and 20 populations of *L*. *ceratophylli*. Average branch lengths are proportional to the number of substitutions per site under a TPM1uF+I substitution model. Posterior probabilities > 0.80 are shown at nodes. Putative cryptic species found using Bayesian Species Delimitation (BSD), *BEAST, Automatic Barcoding Gap Discovery (ABGD), and Single Threshold Generalized Mixed Yule Coalescent models (GMYC) are shown. Abbreviations as in S1 and S2 Tables; independent clonal isolates are indicated by a number (e.g. 01).

In two of the phylogenetic trees (i.e., based on COI gene and 18S rRNA sequences), monophyly of *L*. *melicerta* and *L*. *ceratophylli* was supported (Fig 2 and S1 Fig). Using 18S rRNA sequences, there were only two highly supported clades, one with each species. Additionally, *L*. *melicerta* and *L*. *ceratophylli* co-occurred in Moon Lake, WI. Each clustered with their conspecifics from other habitats based on 18S rRNA and COI gene sequences, supporting monophyly of the two morphospecies. However, ITS sequences did not resolve these taxa (Fig 3). The number of species within *L*. *melicerta* ranged from 9–30 based on COI and 6–45 based on ITS region sequences. For *L*. *ceratophylli*, 3–10 species were identified based on COI gene, and 3–20 based on ITS region sequences (Figs 2 and 3; Table 2). The most conservative results were nine putative species for *L*. *melicerta* (BSD based on COI gene sequences) and three species for *L*. *ceratophylli* (*BEAST based on COI gene sequences). However, *BEAST classified a distinct clade that was represented by a population from Florida as part of species D. That clade was considered a separate species by BSD and ABGD (Fig 2). As *BEAST may have underestimated diversity within *L*. *ceratophylli*, we delimited cryptic species based on BSD analysis of the COI gene sequences, which was the second most conservative method for this species (Table 2).

**Table 2:** Comparison of species delimitation methods.

	*Limnias melicerta*		*Limnias ceratophylli*	
Delimitation method	COI gene	ITS region	COI gene	ITS region
Single threshold GMYC	28	32	8	13
Multi threshold GMYC	25	45	8	20
bGMYC	17	12	3	3
PTP	30	37	10	18
ABGD	14	9	4	3
*BEAST	12	6	3	4
BSD	9	7	4	4

Number of putative cryptic species delimited by seven methods based on partial COI gene and ITS region sequences for *Limnias melicerta* and *L*. *ceratophylli* populations

**Fig 4:**
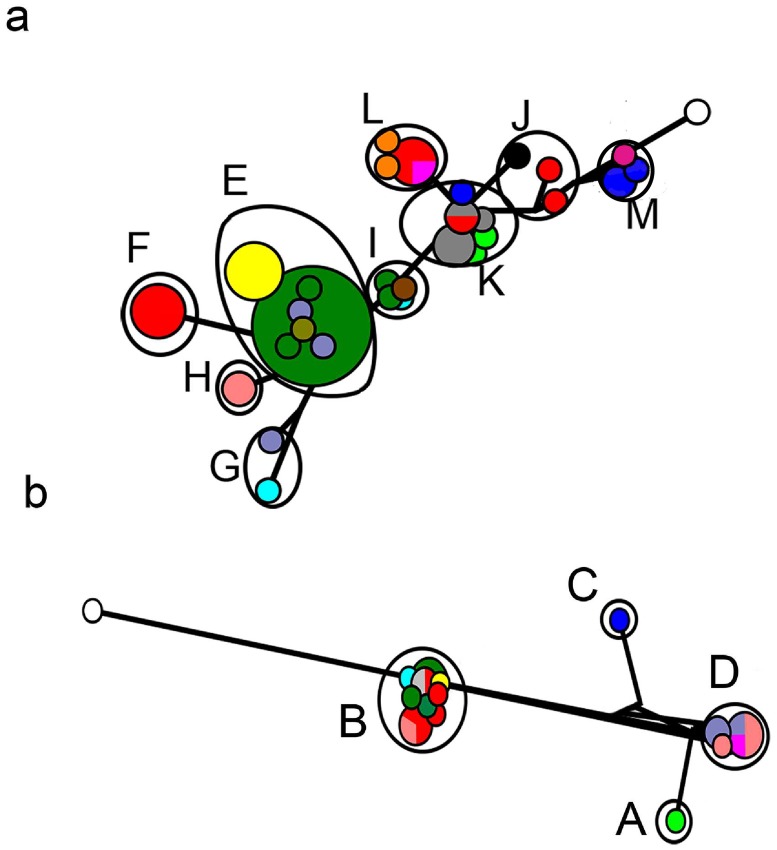
Haplotype network analysis of partial COI gene sequences of a) *Limnias melicerta* and b) *L*. *ceratophylli* populations as determined by the median joining method [79]. Size of circles is proportional to the number of sequences sharing the same haplotype. Branch lengths are proportional to the number of nucleotide substitutions. Black symbols on the network correspond to cryptic species detected by Bayesian Species Delimitation. Color codes are based on the collection site; Texas: red, New Mexico: light green, Georgia: purple, Oregon: light blue, Utah: brown, Oklahoma: gray, California: pink, Florida: dark blue, Wisconsin: dark green, New Hampshire: yellow, Carolina: orange, Pennsylvania: salmon, Minnesota: gold, Australia: dark pink, outgroups: white.

The mean genetic distance in COI gene sequences was 0–15.4% within cryptic species of *L*. *melicerta*, and 0.5–0.6% within cryptic species of *L*. *ceratophylli* (BSD based on COI gene sequences). Among BSD cryptic species, the COI mean genetic distance was 8.1–21.9% for *L*. *melicerta*, and 15.1–21.2% for *L*. *ceratophylli*. For ITS region sequences, within cryptic species mean genetic distance ranged 0.08–2.3% for *L*. *melicerta* and 0.04–1.5% for *L*. *ceratophylli*. Among putative cryptic species, the ITS mean genetic distance was 0–2% for *L*. *melicerta*, and 0–4.3% for *L*. *ceratophylli*. In the haplotype networks based on COI gene sequences for both species, genotype clusters corresponded to the BSD cryptic species (Fig 4).

There was discordance between phylogenetic trees based on COI gene and ITS region sequences. For example, COI lineages E, F, G and H are clustered as one lineage based on the ITS region (lineage 8). In another example, one lineage (lineage 9) based on the ITS region is composed of populations from multiple COI cryptic species (E, I and M) (Figs 2 and 3).

### Isolation by distance

For *Limnias melicerta* populations there was a significant, but weak, correlation between genetic distance and log transformed geographic distance for both markers (Mantel test: COI: r = 0.4 with 95% confidence interval of 0.27–0.46, p < 0.001 and ITS: r = 0.14 with 95% confidence interval of 0.06–0.2, p = 0.03). For *L*. *ceratophylli*, genetic variation in the COI gene was significantly correlated to log transformed geographic distance (Mantel test: r = 0.3; 95% confidence interval of 0.27–0.4, p = 0.001). However, this correlation was not significant based on ITS sequences (Mantel test: r = -0.02; 95% confidence interval of -0.12 to 0.08, p = 0.8).

### Trophi morphology

We obtained 92 SEM images of trophi for *L*. *melicerta* representing six putative species, and 60 images for *L*. *ceratophylli* representing two cryptic species based on COI sequences. Trophi images are available at UTEP Bioinformatics Data Repository. Representative trophi images for each cryptic species are shown in S2 Fig. No significant variation was detected between *L*. *melicerta* and *L*. *ceratophylli* in trophi shape (Discriminant Analysis: **frontal**: Wilks’ Lambda = 1, Chi-square < 0.001, df = 16, p = 1, and **caudal**: Wilks’ Lambda = 1, Chi-square = 0.001, df = 42, p = 1) or trophi size (**frontal**: Mann-Whitney U = 911, p = 0.76, and **caudal**: Mann-Whitney U = 298, p = 0.77). Trophi shape did not show significant variation among putative species of *L*. *melicerta* (Discriminant Analysis: **frontal**: Wilks’ Lambda = 1, Chi-square = 0.001, df = 80, p = 1, and **caudal**: Wilks’ Lambda = 1, Chi-square = 0.0, df = 70, p = 1) or *L*. *ceratophylli* (Discriminant Analysis: **frontal**: Wilks’ Lambda = 1, Chi-square = 0.001, df = 12, p = 1, and **caudal**: Wilks’ Lambda = 1, Chi-square = 0.001, df = 28, p = 1). There was significant variation in trophi size among putative species of *L*. *melicerta* (**frontal**: Kruskal-Wallis Chi-square = 12.2, p = 0.002, and **caudal**: Kruskal-Wallis Chi-square = 10.3, p = 0.02). Cryptic species D had the smallest centroid size (frontal and caudal: 1.33) and cryptic species E had the largest centroid size (frontal: 4, and caudal: 5). However, trophi size showed no significant differentiation among putative species of *L*. *ceratophylli* (**frontal**: Kruskal-Wallis Chi-square = 2.5, p = 0.11, and **caudal**: Kruskal-Wallis Chi-square = 5.9, p = 0.051).

## Discussion

With the advent of molecular tools, detecting cryptic species in rotifers has become a common occurrence, which has improved our knowledge of their diversity. All the previous studies about genetic variation and cryptic species in rotifers are on non-sessile taxa. Here, using two molecular markers we found cryptic diversity within two morphospecies of sessile rotifers, *Limnias melicerta* and *L*. *ceratophylli*. Using the BSD delimitation method, nine putative cryptic species for *L*. *melicerta* and four putative cryptic species for *L*. *ceratophylli* were identified based on COI gene sequences.

Although benefits of using multiple markers to investigate cryptic species is recognized [100–104], most studies of rotifers are based solely on the mitochondrial COI gene (e.g., [24–27,105–107]). In their study of the *Brachionus plicatilis* complex based on all available COI and ITS sequences, Mills *et al*. [29] recommended the nuclear marker ITS1 over COI as it gave a more conservative estimate of species diversity. Similarly, the ITS region was recommended for species delimitation within the *Euchlanis dilatata* complex by Kordbacheh *et al*. [28]. In another study, Papakostas *et al*. [30] reported discordance between COI and ITS markers for species delimitation within the *Brachionus calyciflorus* complex. They argued that species delimitation based on the ITS region better explains morphological variation within the complex. In studies of other taxa, mitochondrial and nuclear markers are concatenated for species delimitation (e.g., bothriurid scorpions *Brachistosternus* spp. complexes [108]; copepod *Cyclops* spp. complexes: [109]), or nuclear markers are used to complement species delimitations which were based on mitochondrial markers (e.g., the sea spider *Pallenopsis patagonica* complex (Hoek, 1881) [110]; Madagascar’s Mouse *Microcebus* spp. complexes [111]). We did not use a concatenated dataset for species delimitation because of differences in coalescent times for these markers [112], and discordance between COI and ITS phylogenetic trees. The observed discordance indicates a lack of coherence within cryptic species for mitochondrial and nuclear markers. This could be due to introgression as was shown for *B*. *calyciflorus* [30] or incomplete lineage sorting as in *E*. *dilatata* [28], *Keratella cochlearis* Gosse, 1851, *Polyarthra dolichoptera* Idelson, 1925, and *Synchaeta pectinata* Ehrenberg, 1832 [113]. In our study, species delimitation based on the ITS region was more conservative as compared to the COI gene. However, the ITS region showed overall low levels of variation (*L*. *melicerta*: ≤3.1%, *L*. *ceratophylli*: ≤4.4%) and failed to separate *L*. *melicerta* and *L*. *ceratophylli* in the phylogenetic analysis. These traditionally recognized species were monophyletic based on COI and 18S rRNA gene sequences. Also, mitochondrial genes can be more informative in the phylogenetic analyses of recently diverged lineages because of their faster rate of evolution [114]. These markers have been successfully used to delimit species in, for example, the Puerto Rican termite *Heterotermes* [115], the copepod *Paracalanus parvus* complex [116], and within several rotifer species (e.g., *K*. *cochlearis* [117]; *S*. *pectinata* [24]; *P*. *dolichoptera* [26]; *E*. *senta* [37]; *Lecane bulla* (Gosse, 1851) [118]). Therefore despite the possibility of over-splitting, we used COI gene sequences for species delimitation within *L*. *melicerta* and *L*. *ceratophylli*.

Populations included in this study were obtained from a wide geographic range. For example, *L*. *melicerta* populations came from Oregon (USA) and the state of Victoria (Australia) a distance of 12,890 km; and *L*. *ceratophylli* populations came from sites spanning from Oregon and Georgia, a distance of 2,726 km. Cryptic species showed various distribution ranges, from a single habitat to several distant habitats. For example, *L*. *melicerta* H was collected only from a permanent lake in Florida, while K was collected from water bodies in New Mexico, Oklahoma, Texas, and Florida. Of the four *L*. *ceratophylli* cryptic species, two were collected from variety of habitats across our sampling range. *L*. *ceratophylli* B was found in sites in Texas, Wisconsin, Oregon, Oklahoma, Pennsylvania, Minnesota, and species D from Georgia, California, and Pennsylvania. The other two *L*. *ceratophylli* cryptic species are singletons, one from a permanent lake in New Mexico, and the other from a pond in Florida. The observed variation in geographic distributions of these putative species may be an artifact of under sampling. The geographic distributions of these cryptic species may be expanded by including samples from additional biogeographical regions or they may represent lineages unique to a given locality. There is also the possibility that some cryptic species are, in fact, cosmopolitan. However, similar patterns in distribution have been reported for other rotifer species complexes such as *E*. *dilatata* (two singletons, five widely distributed species; [28]) and *B*. *plicatilis* (SM7 restricted to North America, SM8 to Australia, and *B*. *ibericus* Ciros-Pérez, Gómez & Serra, 2001 to Europe; [29]). We observed cryptic species represented by few populations that showed wide geographic ranges; as noted above cryptic species G from *L*. *melicerta* was represented by two clonal isolates; one found in Oregon and the other in Georgia, and cryptic species I comprised four populations from Wisconsin, Oregon and Utah. Thus, cryptic species in our study probably vary in their ability for dispersal and colonizing distant habitats. Intraspecific variation in dispersal ability that we infer here has been discussed for other passively dispersing taxa (see [119]).

In our study, genetic variation among populations based on COI gene and ITS region sequences was significantly correlated with geographic distance, except for ITS sequences corresponding to *L*. *ceratophylli* populations. However, because the correlation was weak (or lacking), geographic isolation cannot be considered a strong driver of genetic variation within these cryptic species. Similar to studies on other invertebrates (e.g., 18 invertebrate species [120]; *Daphnia lumholtzi* [121]; *E*. *dilatata* [28]; *S*. *pectinata* [24]; *B*. *calyciflorus* [122]), the populations studied here showed high genetic differentiation across small geographic scales.

We compared the genetic divergence estimates for four sessile rotifer species with non-sessile rotifers. There is evidence of habitat preference in sessile and non-sessile littoral species (e.g., *Collotheca campanulata* [123] and *E*. *dilatata* [44], respectively). As mentioned above, this differentiation can contribute to genetic divergence among populations. Therefore, we expected to see similar levels of genetic differentiation for sessile and littoral morphospecies and lower genetic divergence for planktonic rotifers. Examples of genetic divergence in ITS region and COI gene in rotifer species with different lifestyles are summarized in Table 3. As shown in Table 3, genetic variation in ITS region sequences for littoral rotifer species (*E*. *dilatata* and *L*. *bulla*) was higher than that of planktonic and sessile species complexes. Besides, genetic variation in ITS region in sessile species was similar to that of planktonic groups. It should be noted that there are few instances where the ITS region is used to study rotifer cryptic species and reporting levels of differentiation among cryptic lineages (Table 3). Therefore, there may not be sufficient information to make conclusions about the relationship between rotifer life style and genetic differentiation in the ITS region. On the other hand, higher diversification rates in the COI gene was reported in bdelloid rotifers compared to monogononts [40]. It is hypothesized that this difference is related to the difference in reproductive mode between bdelloids and monogononts (obligatory v. facultative parthenogenic, respectively) [40]. In this study, genetic variation in the COI gene for *L*. *melicerta* and *L*. *ceratophylli* was similar to some other non-rotifer sessile taxa (Table 3). However, genetic variation in the COI gene among rotifer cryptic species was within the same range for planktonic, littoral, and sessile groups (Table 3). A similar pattern has been reported for marine nematodes where genetic structure was not related to their habitat preference (algae versus sediments) [124]. It should be noted that within the superorder Gnesiotrocha, there is no difference in levels of genetic variation between different life styles (at least for the taxa examined thus far; sessile: *Limnias* spp. and *Collotheca* spp. and littoral: *Testudinella clypeata* Müller, 1786, Table 3). All of these rotifers are cyclical parthenogens, which may have contributed to similarity in the range of genetic variation within them. Comparing diversification rate among rotifers with different life history features may not be possible by simply considering the genetic variation in COI gene. To investigate the patterns of diversification for different groups of rotifers, more sophisticated statistical analyses are essential. For example, Fontaneto *et al*. [40] used a statistic measure (γ) to compare the relative position of nodes in the phylogeny to the positions expected under a constant diversification rate scenario. Using this approach, positive values of γ show that diversification rate is higher than expected. γ was used to compare diversification rate among taxa. In another study, Fontanillas *et al*. [125] calculated branch length in the phylogenetic tree and used it to compare diversification rate between sister taxa.

**Table 3:** Genetic divergence (percentage) in ITS region and COI gene sequences of selected planktonic, littoral, and sessile rotifers and some additional sessile invertebrates.

Taxa	Species complex	Life history	ITS region (% variation)	COI gene (% variation)	Reference
Rotifer	*Brachionus plicatilis*	Planktonic	≤1.9 within clades ≥ 2.5 between clades	≤13.3 within clades ≥11.9 between clades	[126]
	*Synchaeta* spp.	Planktonic and littoral	-	0.2–2.7 within clades 5.9–25.3 between clades	[107]
	*Polyarthra dolichoptera*	Planktonic	-	≤4.4 within species ≥5–24 between species	[26]
	*Testudinella clypeata*	Littoral	-	0.16–4.5 within clades 16.7–27.7 between clades	[25]
	*Lecane bulla*	Littoral	0.0–12.5	0.9–16	[118]
	*Euchlanis dilatata*	Littoral	0.0–5.2 within species 1.0–13.4 among species	0.0–18.7 within species 0.2–21.9 among species	[28]
	*Limnias melicerta*	Sessile	0–1.5 within cryptic 0–2 among species	0–11.4 within species 8–20.5 among species	Current study
	*L*. *ceratophylli*	Sessile	0.04–1.5 within species 0–5.3 among species	0.5–0.6 within species 15.1–21 among species	Current study
	*Collotheca campanulata*	Sessile	-	0.0–11.7 within cryptic 10.8–25.2 among species	Unpublished (A. Kordbacheh)
	*C*. *ornata*	Sessile	-	0.3–20.0 within cryptic 14.6–29.0 among species	Unpublished (A. Kordbacheh)
Bryozoan	*Bugula neritina*	Sessile	-	11.5 between two cryptic species	[127]
Tunicate	*Ciona intestinalis*	Sessile	-	11.1–18.4 among lineages	[128]
Porifera	*Cliona celata*	Sessile	-	6.2–8.4 among lineages	[129]

"-" indicates that data were not available.

Sometimes morphological variation among cryptic species is found after a more detailed analysis (e.g., [37,130]). Thus, we investigated trophi morphology to identify potential variation among cryptic species of *L*. *melicerta* and *L*. *ceratophylli*. *Limnias* possess malleoramate trophi. This type of trophi, has large teeth on the rami and thin teeth on the unci [50]. For more detailed descriptions of *Limnias* trophi, see Gosse [59], Meksuwan *et al*. [51] and Wallace *et al*. [54]. While trophi size differed among putative cryptic species of *L*. *melicerta*, it did not show significant variation among putative cryptic species of *L*. *ceratophylli* or between the two. The high variability in trophi size within each morphospecies may have led to failure in detecting significant differences between them. No significant variation in trophi shape was found between *L*. *melicerta* and *L*. *ceratophylli*, or among putative cryptic species. Therefore, trophi shape cannot be used as a diagnostic character in distinguishing them. This morphological conservation and stasis in trophi morphology, despite high genetic variation among putative cryptic species (COI gene: *L*. *melicerta* ≤ 22.7%, *L*. *ceratophylli* ≤ 21.7%), may potentially stem from incongruence between rates of speciation and morphological evolution [131]. Morphological stasis in a variety of traits has been observed in numerous organisms (e.g., the copepod *Eurytemora affinis* (Poppe, 1880) [131]; the butterfly fish *Pantodon buchholzi* Peters, 1876 [132]; two amphipods *Leucothoe ashleyae* Thomas and Klebba, 2006 and *Leucothoe kensleyi* Thomas and Klebba, 2005 [133]). Differences in trophi morphology has been shown to be connected to variation in feeding habits (see Asplanchnidae [134]). Therefore, morphological stasis in trophi could be a result of ecological niche conservatism through similarity in the diet of *L*. *melicerta* and *L*. *ceratophylli*, both of which feed on small particles including yeast [135] and planktonic algae [64]. Morphological stasis should be investigated within morphospecies of other rotifer taxa such as the genus *Floscularia*. This is because, unlike *L*. *melicerta* and *L*. *ceratophylli*, *Floscularia* species show interspecies variation in trophi morphology [136]. Thus, they are likely to show variation in trophi features at the level of cryptic lineages.

Similar to other taxonomic groups, there are a variety of studies that did not find significant morphological variation among rotifer cryptic species. Based on geometric morphometric analyses of lorica and trophi features, Fontaneto *et al*. [130] suggested there are no robust morphological differences between *B*. *plicatilis* and *B*. *manjavacas*. In addition, Leasi *et al*. [25] did not find any significant variation in morphological features of the lorica or body size among seven cryptic species of the *T*. *clypeata* complex. Similarly, morphological characteristics such as variation in lorica size and the presence of a posterior spine were not able to distinguish among eight putative cryptic species of *K*. *cochlearis* [117]. In summary, morphological features have not always been effective in distinguishing among cryptic species of rotifers.

Several studies have complemented molecular methods with other types of data to delimit species boundaries (e.g., [30,106,122,130,137–139]). In this study, we attempted to use a morphological characteristic and geographic isolation of genotypes to find a reliable predictor of genetic variation among putative species within *Limnias melicerta* and *L*. *ceratophylli*. However, trophi shape did not vary among cryptic species, and there was no strong correlation between genetic and geographic distance in these species. Therefore, we did not find sufficient morphological variation or geographic isolation needed to explain the observed genetic differentiation among them. Past studies have focused on ecological differences between *L*. *melicerta* and *L*. *ceratophylli*. For instance, Sarma *et al*. [64] showed that generation time changes in response to food concentration for *L*. *ceratophylli*, while there is no effect on *L*. *melicerta*. While ecological differentiation has been recorded among many rotifer cryptic species (see [140]), to our knowledge there is no information on ecological or behavioral variation among genetic entities of any *Limnias* species. Therefore, we recommend further examination of these features in *Limnias* spp. to elucidate potential mechanisms involved in their speciation. To examine ecological and behavioral differentiation among cryptic species, future studies should include more comprehensive morphological analyses, life table and mating experiments, investigating patterns of metamorphosis and substrate selection by larvae. Studies such as these will aid in examining boundaries of sessile rotifer cryptic species and understanding speciation in rotifers. Moreover, genetic differentiation in other sessile rotifers should be measured to obtain more data for comparing diversification rate among taxa with different life styles (e.g., planktonic versus sessile, colonial versus solitary).

## Supporting information

S1 Table Site and date of collection of *Limnias melicerta* populations and outgroup taxa. GenBank accession numbers for their corresponding partial COI gene, ITS region, and partial 18S rRNA sequences are provided. Number of sequenced clonal lineages and haplotype group(s) for each population are also noted. Missing sequences are specified by “-” for haplotype group and GenBank accession numbers.
(DOCX)

S2 Table Site and date of collection of *Limnias ceratophylli* populations. GenBank accession numbers for their corresponding partial COI gene, ITS region, and partial 18S rRNA sequences are provided. Number of sequenced clonal lineages and haplotype group(s) for each population are also noted. Missing sequences are specified by “-” for haplotype group and GenBank accession numbers. Outgroups are the same as in S1 Table.
(DOCX)

S3 Table Substitution saturation test of molecular markers. Substitution saturation test for partial COI gene, ITS region, and partial 18S rRNA sequences of *Limnias melicerta* and *L*. *ceratophylli* populations implemented in DAMBE v 6. Iss: index of substitution saturation, and Iss.c: critical index of substitution saturation. If Iss is significantly smaller than Iss.c, there is little saturation in the sequences.
(DOCX)

S1 Fig Bayesian inference consensus phylogenetic tree based on partial 18S rRNA sequences of 17 populations of *Limnias melicerta* and 18 populations of *L*. *ceratophylli*. Average branch lengths are proportional to the number of substitutions per site under a JC substitution model. At each node, posterior probabilities > 0.80 are shown. Abbreviations as in S1 and S2 Tables.
(PDF)

S2 Fig Representative trophi from cryptic species of *Limnias melicerta* and *L*. *ceratophylli*. Trophi of cryptic species E, G, I, K, L and M from *L*. *melicerta* and trophi of cryptic species B and D from *L*. *ceratophylli* are shown.
(DOCX)
