# Supplementary material for: Evidence supporting cryptic species within two sessile microinvertebrates, Limnias melicerta and L. ceratophylli (Rotifera, Gnesiotrocha)
Source: PLoS One. 2018 Oct 31;13(10):e0205203. doi: 10.1371/journal.pone.0205203 (PMC6209156; doi:10.1371/journal.pone.0205203)
Supplement: S1 Table — GenBank accession numbers for their corresponding partial COI gene, ITS region, and partial 18S rRNA sequences are provided. Number of sequenced clonal lineages and haplotype group(s) for each population are also noted. Missing sequences are specified by “-” for haplotype group and GenBank accession numbers. (DOCX) [file pone.0205203.s001.docx]

**S1 Table.** **Site and date of collection of *Limnias melicerta* populations and outgroup taxa.**

| Collection Site | Collection date | Abbreviation in text and figures *or* outgroup taxon | GPS coordinates (decimal degrees N/W) | Number of sequenced clonal linages (COI/ITS) | Haplotype groups (COI/ITS) *or* outgroup gene | GenBank accession numbers (COI/ITS/18S) |
| --- | --- | --- | --- | --- | --- | --- |
| Canal Road, Fort Bend Co., TX | 07.30.2013 | L.mel.CRLRd.TX | 29.582778/ -95.539444 | 1/1 | 26/1 | MF787082/ MF787121/ MF795114 |
| Lake Bastrop, Bastrop Co., TX | 08.20.2014 | L.mel.BasLK.TX | 30.133333/ -97.283333 | 1/1 | 24/9 | MF787080/ MF787191/- |
| Cibolo Lake, Guadalupe Co., TX | 08.06.2013 | L.mel.CIB.TX | 28.953056/ -97.873333 | 1/1 | 16/2 | MF787067/ MF787133/- |
| Inks Lake State Park, Bastrop Co., TX | 08.20.2014 | L.mel.INK.TX | 30.730596/ -98.383275 | 1/1 | 17/9 | MF787070/ MF787190/- |
| Mescalero Canyon, El Paso Co., TX | 11.24.2014 | L.mel.EHT.TX | 31.9188166/ -06.0403666 | 5/4 | 8/3 | MF787054-8/ MF787137, MF787153, MF787155, MF787157/ MF795101 |
| Rio Grande, American Dam, El Paso Co., TX | 12.10.2013 | L.mel.AMD.TX | 31.784234/ -106.527845 | 2/1 | 17/8 | MF787068-9/ MF787189/ MF795106 |
| Rattlesnake Springs, Carlsbad Caverns National Park, Eddy Co., NM | 07.08.2014 & 10.17.2014 | L.mel.RAT.NM | 32.1097/ -104.471625 | 2/3 | 20 & 21/1 | MF787074-5/ MF787122-3 & MF787174/ MF795102 |
| Balboa Park Natural Area, Los Angeles Co., CA | 02.29.2016 | L.mel.Balboa.CA | 34.1764278/ -18.4729139 | 1/1 | 17/7 | MF787071/ MF787188/- |
| Glassman pond, Weber Co., UT | 05.20.2016 | L.mel.Glass.UT | 41.179735/ -111.951601 | 1/1 | 15/3 | MF787066/ MF787183/ MF795110 |
| Timber Lake Ditch, Mount Hood National Forest, Clackamas Co., OR | 05.25.2014 | L.mel.TIM.OR | 45.083424/ -122.050234 | 1/1 | 12/5 | MF787087/ MF787178/ MF795103 |
| Devil’s Lake Roadside Ditch, Lincoln Co., OR | 05.24.2014 | L.mel.DEV.OR | 44.967222/ -124.016667 | 0/1 | -/3 | -/MF787134/ MF795098 |
| Devil’s Lake, Devil’s Lake State Park, Lincoln Co., OR | 05.24.2014 | L.mel.DEVD.OR | 44.970664/  -124.012335 | 1/1 | 9/3 | MF787059/ MF787135/ MF7950100 |
| Minto Park pond, Marion Co., OR | 05.25.2014 | L.mel.MIN.OR | 44.919914/ -123.060946 | 0/1 | -/3 | -/MF787136/- |
| Flint pond, Hillsborough Co., NH | 11.15.2015 | L.mel.FLNT.NH | 42.74926/ -71.54951 | 7/9 | 5/3 | MF787040 & MF787045-50/ MF787150-2, MF787154, MF787156, MF787158-61/ MF795112 |
| Boxford pond, Essex Co., MA | 07.16.2017 | L.mel.BOX.MA | 42.673355/ -71.022617 | 0/1 | -/3 | -/MF787184/- |
| Mascuppie Lake, Middlesex Co., MA | 07.10.2017 | L.mel.MAS.MA | 42.672556/ -71.384359 | 1/1 | 30/3 | MH400074/ MH411244/- |
|  |  |  |  |  |  |  |
| Purgatory Creek Wetlands Park, Hennepin Co., MN | 07.10.2017 | L.mel.Purg.MN | 44.856886/ -93.44072 | 0/1 | -/11 | -/MH411246/- |
| Red Rock Lake, Hennepin Co., MN | 07.10.2017 | L.mel.RedRock.MN | 44.843811/ -93.470554 | 0/1 | -/3 | -/MH411243/- |
| Moon (Birch) Lake, Marquette Co., WI | 09.28.2014 & 10.05.2014 | L.mel.MN.WI | 43.806367/ -89.366509 | 25/19 | 2/3 | MF787018-39 & MF787041-3/ MF787138-49, MF787162-3 & MF787169-73/ MF795099 |
| White River Marsh Wildlife Area, Marquette Co., WI | 07.06.2015 | L.mel.WRW.WI | 43.925724/ -89.099011 | 1/2 | 13/5 | MF787064/ MF787180-1 |
| Minister Lake, Racine Co., WI | 08.09.2015 | L.mel.MinLK.WI | 44.54277/ -89.26954 | 1/1 | 14/3 | MF787065/ MF787182/- |
| Carolina pond, James Island, Charleston Co., SC | 10.22.2015 | L.mel.SC | 32.7084/ -79.9518 | 2/3 | 18 & 19/6 | MF787072-3/ MF787185-7/- |
| Crescent Lake, Beaver Co., OK | 06.25.2015 | L.mel.CRS.OK | 33.927829/-97.312675 | 3/4 | 22, 23, & 24/1 | MF787076 & MF787078-9/MF787126 & MF787128-30/ MF795113 |
| Statue pond, Beaver Co., OK | 06.25.2015 | L.mel.STA.OK | 33.930405/ -97.3103 | 1/3 | 3/1 | MF787077/ MF787127 & MF787131-2/ MF795105 |
| Lake Niskey, Gwinnett Co., GA | 04.16.2016 | L.mel.Niskey.GA | 33.7155/ -84.5311667 | 1/1 | 10/3 | MF787060/ MF787164/ MF795104 |
| Canary Lake, Gwinnett Co., GA | 04.17.2016 | L.mel.CanLK.GA | 33.9771667/ -84.1371667 | 1/1 | 6/3 | MF787051/ MF787165/ MF795107 |
| Berkeley View, Gwinnett Co., GA | 04.18.2016 | L.mel.BeView.GA | 33.99025/ -84.1623833 | 1/1 | 1/3 | MF787017/ MF787166/- |
| Lauren pond, Gwinnett Co., GA | 04.18.2016 | L.mael.Lauren.GA | 33.9371667/ -84.13575 | 1/1 | 4/3 | MF787044/ MF787167/- |
| Street pond, Gwinnett Co., GA | 04.18.2016 | L.mel.STP.GA | 33.9360833/ -84.1366 | 1/1 | 7/3 | MF787052/ MF787168/- |
| Fish Camp, Highlands Co., FL | 01.09.2015 | L.mel.FishCP.FL | 25.941935/ -80.441761 | 3/3 | 11/4 | MF787061-3/ MF787175-7/ MF795109 |
| Krome pond, Miami-Dade Co., FL | 01.09.2015 | L.mel.KRM.FL | 25.883615/ -80.484920 | 1/1 | 28/10 | MF787083/ MF787192/- |
| Site 4, Miami-Dade Co., FL | 01.09.2015 | L.mel.SIT4.FL | 25.761889/ -80.50241 | 1/1 | 29/5 | MF787085/ MF787179/- |
| Lake Jackson, Highlands Co., FL | 01.09.2015 | L.mel.JAC.FL | 27.48775/ -81.47664 | 1/1 | 25/1 | MF787081/ MF787124/ MF795111 |
| Lake Okeechobee, Glades Co., FL | 01.09.2015 | L.mel.LKO.FL | 26.830456/ -80.941303 | 1/1 | 29/1 | MF787084/ MF787125/ MF795108 |
| Ryans Lagoon Nature Conservation Reserve | - | L.mel.Murray.Australia | -6.110722/ 146.966644 | 1/1 | 31/12 | MH400073/ MH411245/- |
| Glassman pond, Weber Co., UT | 05.20.2016 | *Collotheca campanulata* | 41.179735/ -111.951601 |  | outgroup for *18S rDNA* | -/-/MF795133 |
| Steep Hill Creek, Natchitoches, LA | 03.13.2014 | *Ptygura pilula* | 31.466865/-93.100005 |  | outgroup for *COI* gene | MG255828/-/- |
| Buffalo Lake, Marquette Co., WI | 12.08.2013 | *Sinantherina socialis* | 43.7738/  -89.4092 |  | outgroup for *COI* gene | MG255829/-/- |
| Moon (Birch) Lake, Marquette Co., WI | 09.28.2014 | *Ptygura brachiata* | 43.806367/ -89.366509 |  | outgroup for ITS region | -/MH492676/- |
| Boxford pond, Essex Co., MA | 07.16.2017 | *Floscularia conifera* | 42.673355/ -71.022617 |  | outgroup for ITS region | -/MG263749/- |

GenBank accession numbers for their corresponding partial COI gene, ITS region, and partial 18S rRNA sequences are provided. Number of sequenced clonal lineages and haplotype group(s) for each population is also noted. Missing sequences are specified by “-“ for haplotype group and GenBank accession numbers.
