# Supplementary material for: Evidence supporting cryptic species within two sessile microinvertebrates, Limnias melicerta and L. ceratophylli (Rotifera, Gnesiotrocha)
Source: PLoS One. 2018 Oct 31;13(10):e0205203. doi: 10.1371/journal.pone.0205203 (PMC6209156; doi:10.1371/journal.pone.0205203)
Supplement: S2 Table — GenBank accession numbers for their corresponding partial COI gene, ITS region, and partial 18S rRNA sequences are provided. Number of sequenced clonal lineages and haplotype group(s) for each population are also noted. Missing sequences are specified by “-” for haplotype group and GenBank accession numbers. Outgroups are the same as in S1 Table. (DOCX) [file pone.0205203.s002.docx]

**S2 Table.** **Site and date of collection of *Limnias ceratophylli* populations.**

| Collection Site | Collection date | Abbreviation in text and figures | GPS coordinates (decimal degrees N/W) | Number of sequenced clonal linages (COI/ITS) | Haplotype group (COI/ITS) | GenBank accession numbers (COI/ITS/18S) |
| --- | --- | --- | --- | --- | --- | --- |
| Lake Worth, Tarrant Co., TX | 08.19.2013 | L.cer.Worth.TX | 32.810833/ -97.4325 | 2/4 | 2&3/1 | MF786994-5/ MF787088-91/ MF795127 |
| Lake Waco Wetlands, McLennan Co., TX | 12.10.2012 | L.cer.Waco.TX | 31.5836/ -97.2006 | 1/1 | 3/3 | MF786997/ MF787120/ MF795128 |
| Feather Lake Wildlife Sanctuary, El Paso Co., TX | 09.01.2016 | L.cer.FL.TX | 31.6890972/ -06.305266 | 1/1 | 7/3 | MF787005/ MF787193/ MF795130 |
| Sam Rayburn Reservoir, Sabine Co., TX | 07.30.2013 | L.cer.SAM.TX | 31.061244/ -94.106127 | 1/1 | 6/3 | MF787003/ MF787093/- |
| Morphy Lake, Mora Co., NM | 06.10.2016 | L.cer.Morphy.NM | 35.941042/ -05.396167 | 1/1 | 1/2 | MF786993/ MF787092/ MF795115 |
| Echo Lake, El Dorado Co., CA | 02.29.2016 | L.cer.ECH.CA | 34.0705/ -18.260663 | 1/1 | 13/3 | MF787012/ MF787109/ MF795132 |
| Burriston pond, Juab Co., UT | 05.20.2016 | L.cer.Burriston.UT | 39.796162/ -11.865922 | 0/1 | -/4 | -/MF787118/  MF795121 |
| Blue Lake, Multnomah Co., OR | 05.25.2014 | L.cer.Blue.OR | 45.5565/ -122.4481 | 1/1 | 5/3 | MF787002/ MF787094/ MF795118 |
| Flint pond, Hillsborough Co., NH | 04.30.2014 | L.cer.FLNT.NH | 42.74926/ -71.54951 | 0/1 | -/4 | -/MF787112/ MF795126 |
| Naticook Lake, Hillsborough Co., NH | 07/16/2016 | L.cer.NAT.NH | 42.8200/ -71.5257 | 0/1 | -/3 | -/MF787111/  MF795119 |
| Dragonfly pond, Hennepin Co., MN | 07.10.2017 | L.mel.Dragonfly.MN | 44.879451/ -93.683848 | 1/1 | 8/3 | MH400072/ MH411242/- |
| The Fishing Pond, Nockamixon State Park, Bucks Co., PA | 05.20.2016 | L.cer.NockFS.PA | 40.472567/ -75.224823 | 1/1 | 13/4 | MF787013/ MF787117/ MF795120 |
| Lake Nockamixon, Nockamixon State Park, Bucks Co., PA | 05.20.2016 | L.cer.NockSP.PA | 40.462634/ -75.232702 | 1/1 | 13/11 | MF787011/ MF787119/ MF795123 |
| Twin Lakes, Lower Lake, Westmoreland Co., PA | 08.06.2012 | L.cer.TLL.PA | 41.8571827/ -5.3366041 | 1/0 | 3/- | MF786996/-/- |
| Gothic Mill pond, Fond Du Lac Co., WI | 06.27.2016 | L.cer.GMP.WI | 43.8427606/ -8.8301113 | 1/1 | 9/3 | MF787006/ MF787110/- |
| Moon (Birch) Lake, Marquette Co., WI | 06.08.2015 & 09.13.2015 | L.cer.MN.WI | 43.806367/ -89.366509 | 5/12 | 4 & 10/3 | MF786998-7001 &  MF787007/ MF787096 &  MF787098-108/  MF795117 |
| Lake Mendota, Dane Co., WI | 07.22.2014 | L.cer.MEN.WI | 43.0789083/ -9.4192250 | 1/1 | 11/3 | MF787086/ MF787095/ MF795129 |
| Duck pond, Beaver Co., OK | 11.24.2013 | L.cer.DUC.OK | 35.551944/ -97.574722 | 1/1 | 7/3 | MF787004/ MF787097/ MF795125 |
| Piedmont Park Lake, Gwinnett Co., GA | 04.16.2016 | L.cer.Pied.GA | 33.78605/ -4.3722667 | 1/1 | 13/4 | MF787010/ MF787114/- |
| Hi Loch Lomond, Fulton Co., GA | 04.16.2016 | L.cer.HiLoch.GA | 33.7121167/ -84.545 | 1/1 | 14/4 | MF787014/ MF787116/ MF795116 |
| Norman Lake, Gwinnett Co., GA | 04.17.2016 | L.cer.NorLK.GA | 33.996/ -4.1287167 | 1/1 | 15/4 | MF787016/ MF787115/ MF795122 |
| East Point Reservoir, Fulton Co., GA | 04.17.2016 | L.cer.EastPoint.GA | 33.9360833/ -4.1366000 | 1/0 | 14/- | MF787015/-/ MF795131 |
| Harold A Campbell pond, Walton Co., FL | 11.10.2015 | L.cer.HCP.FL | 26.338031/ -80.628171 | 1/1 | 12/4 | MF787009/ MF787113/ MF795124 |

GenBank accession numbers for their corresponding partial COI gene, ITS region, and partial 18S rRNA sequences are provided. Number of sequenced clonal lineages and haplotype group(s) for each population are also noted. Missing sequences are specified by “-“ for haplotype group and GenBank accession numbers. The outgroup taxa are the same as in S1 Table.
