# Supplementary material for: Evidence supporting cryptic species within two sessile microinvertebrates, Limnias melicerta and L. ceratophylli (Rotifera, Gnesiotrocha)
Source: PLoS One. 2018 Oct 31;13(10):e0205203. doi: 10.1371/journal.pone.0205203 (PMC6209156; doi:10.1371/journal.pone.0205203)
Supplement: S3 Table — Substitution saturation test for partial COI gene, ITS region, and partial 18S rRNA sequences of Limnias melicerta and L. ceratophylli populations implemented in DAMBE v 6. Iss: index of substitution saturation, and Iss.c: critical index of substitution saturation. If Iss is significantly smaller than Iss.c, there is little saturation in the sequences. (DOCX) [file pone.0205203.s003.docx]

**S3 Table. Substitution saturation test of molecular markers.**

| Marker | Iss | Iss.c | T | df | p |
| --- | --- | --- | --- | --- | --- |
| COI gene | 0.26 | 0.80 | 27.27 | 619 | <0.001 |
| ITS region | 0.11 | 0.74 | 60.17 | 687 | <0.001 |
| 18S *rRNA* | 0.01 | 0.75 | 237.46 | 810 | <0.001 |

Substitution saturation test for partial COI gene, ITS region, and partial 18S rRNA sequences of *Limnias melicerta* and *L. ceratophylli* populations implemented in DAMBE v 6. Iss: index of substitution saturation, and Iss.c: critical index of substitution saturation. If Iss is significantly smaller than Iss.c, there is little saturation in the sequences.
