# Supplementary figures and images for: Evidence supporting cryptic species within two sessile microinvertebrates, Limnias melicerta and L. ceratophylli (Rotifera, Gnesiotrocha)
Source: PLoS One. 2018 Oct 31;13(10):e0205203. doi: 10.1371/journal.pone.0205203 (PMC6209156; doi:10.1371/journal.pone.0205203)

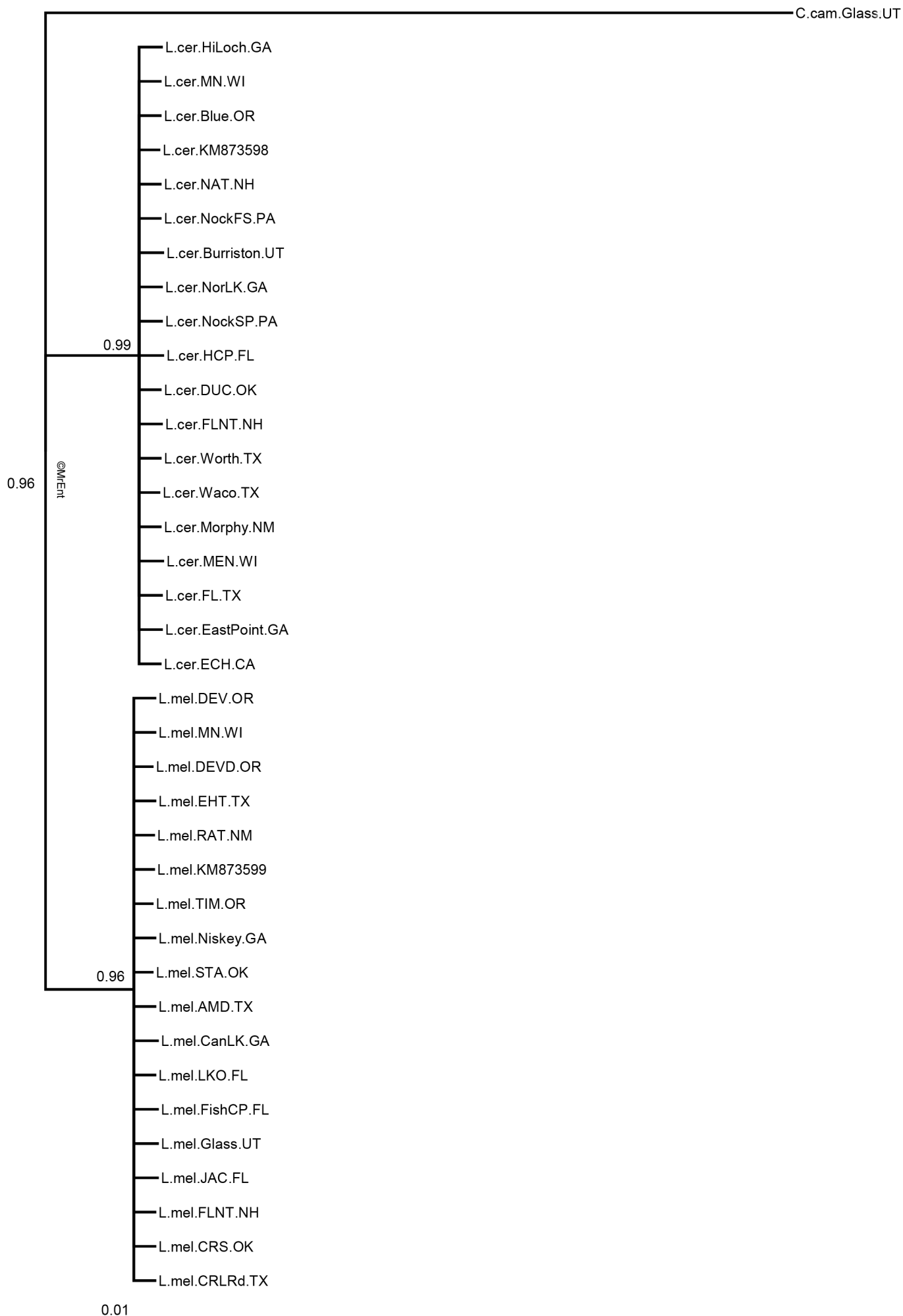

Supplement: S1 Fig — Average branch lengths are proportional to the number of substitutions per site under a JC substitution model. At each node, posterior probabilities > 0.80 are shown. Abbreviations as in S1 and S2 Tables. (PDF) [file pone.0205203.s004.pdf]
