# Supplementary material for: Evidence supporting cryptic species within two sessile microinvertebrates, Limnias melicerta and L. ceratophylli (Rotifera, Gnesiotrocha)
Source: PLoS One. 2018 Oct 31;13(10):e0205203. doi: 10.1371/journal.pone.0205203 (PMC6209156; doi:10.1371/journal.pone.0205203)
Supplement: S2 Fig — Trophi of cryptic species E, G, I, K, L and M from L. melicerta and trophi of cryptic species B and D from L. ceratophylli are shown. (DOCX) [file pone.0205203.s005.docx]

**S2 Fig. Representative SEM images of trophi from cryptic species of *Limnias melicerta* and *L. ceratophylli*.** Trophi of cryptic species E, G, I, K, L and M from *L. melicerta* and trophi of cryptic species B and D from *L. ceratophylli* are shown.


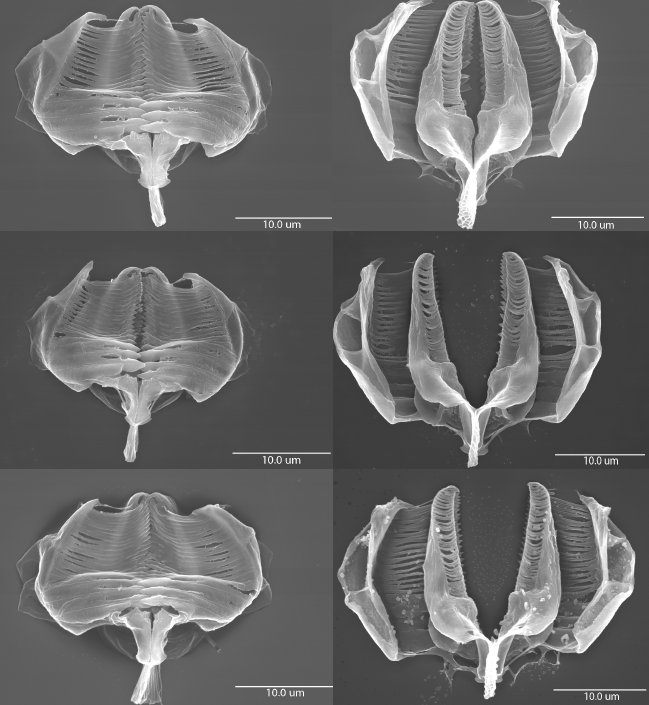


**S2.1 Fig. Trophi of *Limnias melicerta*.** Cryptic species E, frontal view (top left), caudal view (top right); Cryptic species G, frontal view (middle left), caudal view (middle right); Cryptic species I, frontal view (bottom left), caudal view (bottom right).


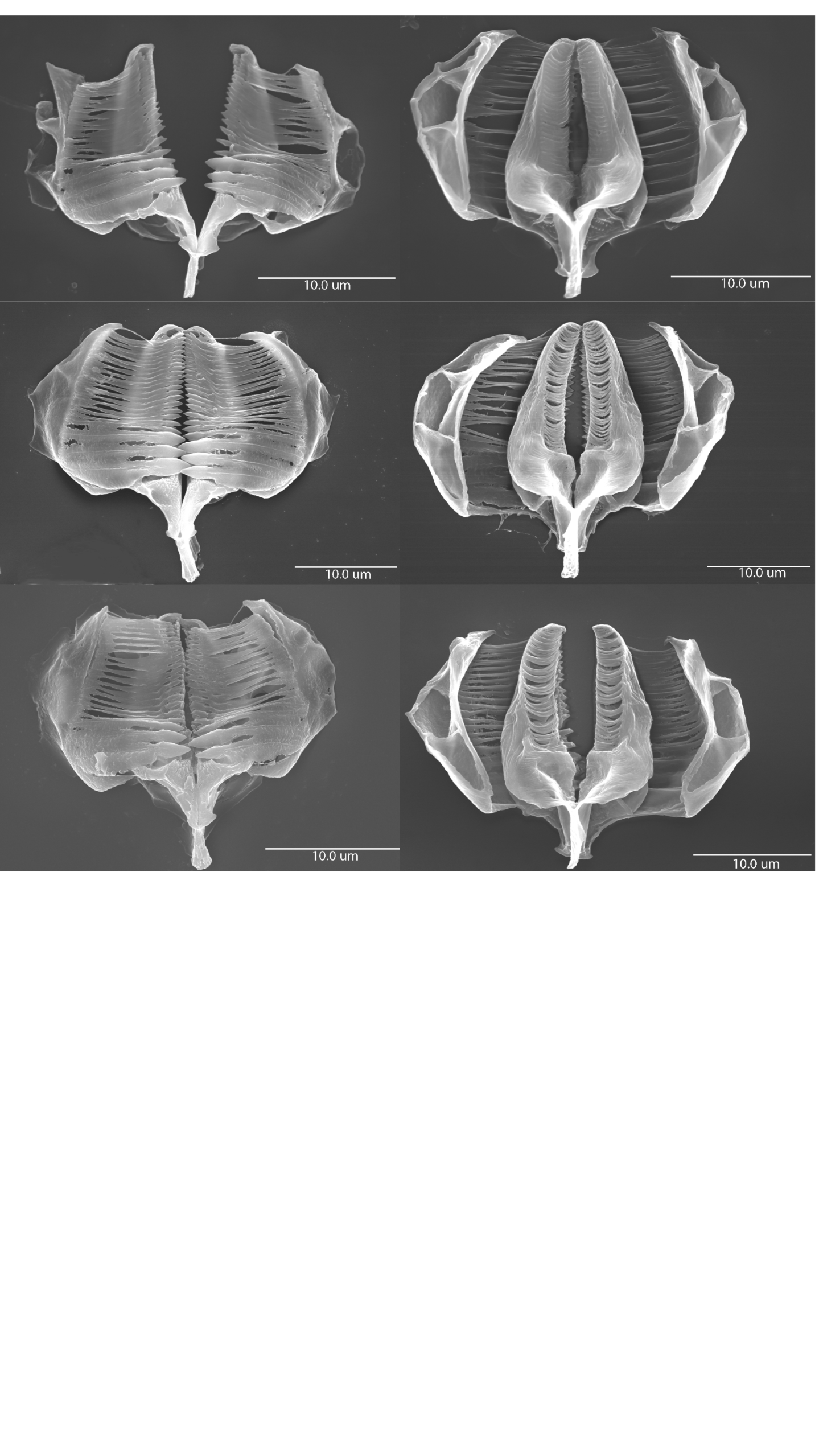


**S2.2 Fig. Trophi of *Limnias melicerta*.** Cryptic species K, frontal view (top left), caudal view (top right); Cryptic species L, frontal view (middle left), caudal view (middle right); Cryptic species M, frontal view (bottom left), caudal view (bottom right).


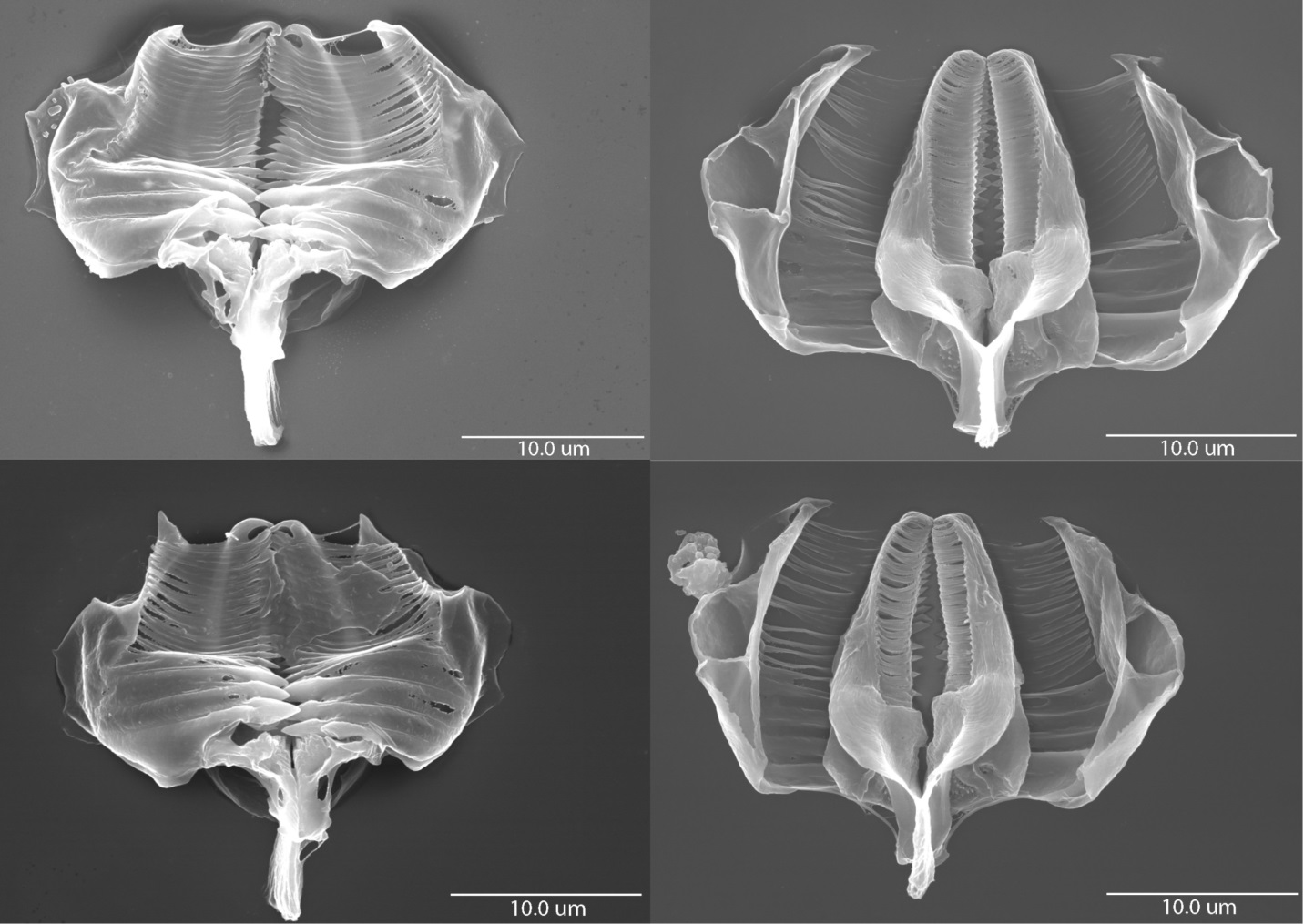


**S2.3 Fig. Trophi of *Limnias ceratophylli*.** Cryptic species B, frontal view (top left), caudal view (top right); Cryptic species D, frontal view (bottom left), caudal view (bottom right).
